# Binge drinking indirectly predicts a negative emotional memory bias through coping motivations and depressive symptoms: The role of sex/gender

**DOI:** 10.3389/fpsyg.2022.998364

**Published:** 2022-11-21

**Authors:** Samantha Johnstone, Kesia Courtenay, Todd A. Girard

**Affiliations:** Department of Psychology, Toronto Metropolitan University, Toronto, ON, Canada

**Keywords:** alcohol, binge drinking, emotional memory, depressive symptoms, coping motivations, sex factors

## Abstract

**Background:**

In this three-part study, we investigate whether the associations between binge and problematic drinking patterns with a negative emotional memory bias (NMB) are indirectly related through coping motivations and depressive symptoms. We also address potential sex differences in these relations.

**Methods:**

Participants (*N* = 293) completed the Timeline Followback to assess binge drinking, the Alcohol Use Disorder Identification Test (AUDIT) to assess problematic alcohol use, the Drinking Motives Questionnaire-Revised to assess coping motivations, and the Depression, Anxiety, and Stress Scales-21 to assess depression. Participants were asked to identify whether 30 emotional sentences were self-referent or not in an incidental encoding task; 24 h later they were asked to recall as many sentences as possible and a negative memory bias score was calculated.

**Results:**

Across all three studies, we found significant bivariate relations between AUDIT scores, coping, depression, and an NMB, particularly for sentences participants deemed self-referent. In two undergraduate samples, there were significant indirect effects through coping motivations and depressive symptoms between binge drinking and an NMB in females as well as between AUDIT scores and an NMB in females only. In the community sample, there was only an indirect effect through coping motives, but this was observed in both females and males.

**Conclusion:**

These findings support a relation between binge drinking as well as problematic alcohol use and a self-referent NMB in the context of coping motivations for alcohol use and depressive symptoms. Moreover, the pattern of findings suggests this model primarily holds for females, yet may also apply to males at higher levels of problematic alcohol use.

## Introduction

Psychopathology-congruent memory biases are a purported cognitive mechanism underlying mental health conditions and maladaptive behaviors (Blaut et al., 2013; Zupan et al., 2017; Carbia et al., 2020). For instance, the occurrence of a negative emotional memory bias (NMB), defined as the propensity to preferentially recall negative information (Duyser et al., 2020), has recently been associated with frequent binge drinking (BD) in female undergraduate students (Carbia et al., 2020). However, not everyone who BDs frequently shows an NMB, which begs the question, what sets apart those who do? In this vein, the NMB is potentially indicative of emotional dysregulation, seen through increased attention to and identification with negative stimuli (see Disner et al., 2011). Emotional dysregulation is also a central feature of the negative reinforcement model of addiction (Koob, 2013), which posits that repeated heavy alcohol consumption serves to relieve unpleasant states (e.g., negative mood). Thus, the NMB may be more likely to occur among individuals for whom this model is most salient, whether in the form of BD or other problematic alcohol use.

Importantly, contemporary conceptualizations of the negative reinforcement model propose that individuals who engage in excessive drinking behavior tend to endorse coping with negative affect as a motivation for alcohol use (Webb et al., 2020), and report greater depressive symptoms (Keough et al., 2015), which by extension, may also relate to an NMB. Indeed, greater alcohol consumption is associated with using alcohol as a coping mechanism (Patrick and Schulenberg, 2011; Decaluwe et al., 2019), which may facilitate alcohol-related neurological changes that, in turn, increase recall of negative information (Stephens and Duka, 2008). Additionally, greater alcohol consumption and drinking-related consequences are related to depression (Kuria et al., 2012; Connell et al., 2015) which is further associated with the NMB (Bradley and Mogg, 1994; Blaut et al., 2013; Zupan et al., 2017). Thus, given the relations among excessive alcohol use with coping motivations and depressive symptoms, here, we investigate the potential intervening roles of coping motivations and depressive symptoms on the relation between BD, more general problematic alcohol use, and the NMB.

### Problematic drinking and a negative emotional memory bias

Over time, excessive alcohol consumption is associated with structural brain changes (Carpenter-Hyland and Chandler, 2007; Spear, 2018) in regions that play key roles in encoding and retrieving emotional memories (Tyng et al., 2017). To elaborate, the pattern of intoxication-withdrawal characteristic of repeated BD episodes may dysregulate the amygdala and prefrontal cortex (Stephens and Duka, 2008). Related reductions in prefrontal-mediated inhibitory control coupled with amygdala hyperactivity may thus predispose individuals who BD frequently to preferentially attend to and recall negative information (Stephens and Duka, 2008; Tolomeo et al., 2021). Importantly, despite differing patterns of use, young adults who BD frequently exhibit cognitive and emotional profiles similar to those observed in repeatedly detoxified individuals with prolonged alcohol use disorders (Duka et al., 2004; Stephens and Duka, 2008). Thus, it is prudent to understand the NMB in the context of BD and problematic drinking more generally (Disner et al., 2011).

Despite longstanding investigations of the NMB in conditions involving emotional dysregulation (Bradley and Mogg, 1994; Disner et al., 2011; Duyser et al., 2020), its relation to alcohol use remains relatively understudied. In a recent exception, Carbia et al. (2020) observed that higher frequencies of BD episodes among female (but not male) college students predicted greater recall of negative compared to neutral words across immediate learning trials and after a 30-min delay. However, given that emotional stimuli generally tend to elicit superior recall relative to neutral stimuli (Tyng et al., 2017), it is also important to establish preferential recall of negative relative to positive stimuli when determining the presence of an NMB. In addition, longer delays (≥ 1 day) are often more sensitive to observing explicit memory biases, which are thought to reflect time-dependent processes of emotional effects on memory consolidation. Here, we investigate the above association, extending the delay to 1 day, and explore relations of the NMB with more general problematic alcohol use as well as assess potential mediating factors: Namely, use of alcohol to cope and symptoms of depression.

### Role of coping motivations for alcohol use

Coping motivations for alcohol use (i.e., using alcohol to relieve negative affect) may contribute to the association between alcohol consumption and the NMB. Indeed, endorsement of coping motivations underlying alcohol consumption is associated with greater frequency of BD episodes, greater quantities of alcohol consumed per episode (Stewart et al., 2011; Decaluwe et al., 2019), as well greater cumulative years of BD (Patrick and Schulenberg, 2011). All such factors may lead to more pronounced neurological consequences that may render individuals more vulnerable to the NMB. Furthermore, coping motivations are associated with implicit memory associations formed while drinking in a negative affective state. For example, coping motivation is related to an alcohol-related interpretative bias reflected by a tendency to identify external negative situations as being contextually related to alcohol (Salemink and Wiers, 2014). Similarly, repeated drinking during periods of negative affect may result in a learned association between internally “feeling bad” and cravings for alcohol (Zack et al., 1999, 2002). Thus, certain external triggering situations and internal negative affect may cue a craving for alcohol in those who drink to cope, thereby perpetuating a negative reinforcement cycle (Zack et al., 2002; Cox et al., 2006; Kessler et al., 2013). As such, elicitation of alcohol cravings in negative situations and increased consumption as a function of coping motivations may also explain the pathway from problematic drinking to an NMB.

### Role of depression

The co-occurrence of depression is posited to exacerbate the emotional and cognitive consequences of excessive alcohol use (Connell et al., 2015). For example, the biased recall of alcohol-related stimuli accompanying alcohol dependence is stronger in those with comorbid depression (Fridrici et al., 2014). Furthermore, depressed individuals high on BD measures attend to negative stimuli more quickly compared to those who are either nondepressed or do not BD as often (Connell et al., 2015). As previously noted, depression is also linked to an NMB (Disner et al., 2011; Duyser et al., 2020), that is, especially pronounced for self-referent information (Bradley and Mogg, 1994; Taylor and John, 2004; Zupan et al., 2017). This “self-NMB” may result from pervasive negative self-views in depression that increase the salience of negative information referent to self-concept, rendering it more competitive for retrieval (Disner et al., 2011, 2017; Zupan et al., 2017). The NMB is further proposed to relate to difficulty disengaging from negative information, thus perpetuating negative self and world views, two central features of depression (Beck, 1967; Disner et al., 2011). As such, we propose that depression plays a role in the path from excessive alcohol use to an NMB for self-referent information.

### Current aims

In sum, individuals who engage in problematic forms of drinking are at risk of developing an NMB. Moreover, this relation may be explained by intervening relations with coping motivations and depressive symptoms. Here, we fit a serial multiple mediation model to assess whether greater alcohol use predicts a tendency to use alcohol to cope with negative emotions (negative reinforcement), thereby predicting depressive symptoms due to excessive drinking and emotional dysregulation, and ultimately perpetuating an NMB. Further, we expect that the NMB will be most pronounced for sentences deemed self-referent as is seen in depression (Disner et al., 2011). We recognize that the aforementioned factors might act in a reciprocal manner, thus resulting in a feedback loop, and as such fit other possible models to assess the relative fit of our hypothesized predictive path.

We first explore this model with a female undergraduate sample to assess indirect effects in an extension of the sex-specific findings reported by Carbia et al. (2020). Study 2 then aims to replicate this in a sample of male and female undergraduate students to assess potential sex/gender differences in the aforementioned model. Finally, Study 3 assesses the generalizability of findings to a community sample.

## Study 1

### Methods

#### Participants

We recruited 62 female participants through an undergraduate psychology research pool; 5 were excluded from analyses due to missing data and 3 were excluded due to consuming alcohol on Day 1 (i.e., were at risk of being intoxicated during testing). Sample characteristics of the remaining 54 participants are presented in Table 1. We engaged with participants *via* secure videoconferencing (Zoom Video Communications, Inc.) for course credit; cameras were off during self-report portions to minimize undue social influence. The Toronto Metropolitan University REB approved the project (Studies 1–3) in accordance with the Canadian Tri-Council Policy Statement (TCPS2) on ethical conduct for research involving humans and all participants (Studies 1–3) provided informed consent.

**Table 1:** Sample characteristics across the three studies.

	**Study 1 Student**	**Study 2 Student**		**Study 3 Community**	
	**Female**	**Female**	**Male**	**Female**	**Male**
n	54	94	50	47	48
**Age**	23^bc^(7.1)	21.5^c^ (7.3)	23.6^bc^ (7.4)	25.3 ^ab^(4.9)	27.4^a^ (5.0)
**Ethnicity (%)**					
White/European	52	39	40	45	52
South/East Asian	22	34	30	32	19
Middle Eastern	9	4	14	6	8
Black	7	9	4	13	19
Hispanic	2	5	0	4	2
Multiracial	7	10	12	0	0
**AUDIT**	6.9^b^ (5.9)	4.6^b^ (4.5)	7.7^b^ (5.9)	6.4^b^ (6.9)	12.2^a^ (10.1)
**BD episodes**	2.3 (4.4)	2.8 (6.4)	3.9 (6.0)	1.8 (4.2)	1.0 (1.8)
**DMQ-R**					
Coping	10.9^b^ (5.3)	9.4^b^ (5.4)	10^b^ (4.8)	10.8^b^ (5.1)	12.6^a^ (5.5)
Enhancement	13.2 (5.3)	12.2 (5.1)	13.8 (5.1)	11.8 (5.3)	14.6 (6.8)
Social	16.1 (5.4)	14.9 (5.8)	16.2 (5.3)	13.4 (5.1)	14.4 (5.7)
Conformity	8.4^b^ (3.9)	8^b^ (3.7)	8.2^b^ (4)	9.4^b^ (5)	12^a^ (4.7)
**DASS-21**					
Depression	14.4^a^ (10.1)	12.7^a^ (9.8)	12.8^a^ (10.5)	10.7^b^ (9.3)	10.6^b^ (7.2)
Anxiety	15.3 (10.6)	11.5 (9.6)	10.3 (8.8)	9.8 (9)	11 (7.9)
Stress	18.9^a^ (9.5)	14.3^b^ (9.3)	14.6^b^ (9.8)	13.7^b^ (10.1)	13.2^b^ (7.6)
**Recall Performance**					
Total Recall	1.78 (1.46)	3.68 (2.70)	2.76 (2.18)	2.98 (2.6)	3.44 (2.31)
**Negative**					
Self	0.32^b^ (0.56)	0.66^a^(0.96)	0.38^b^ (0.64)	0.30^b^ (0.51)	0.19^b^ (0.45)
Not-Self	0.56 (0.72)	0.90 (1.15)	0.78 (1.10)	0.74 (1.03)	0.96 (1.09)
**Positive**					
Self	0.72^b^ (0.79)	1.61^b^(1.44)	1.1^b^ (1.04)	1.66^b^ (1.78)	2.17^a^ (1.63)
Not-Self	0.17 (0.52)	0.51 (0.90)	0.50 (0.93)	0.28 (0.62)	0.12 (0.33)

Data represent Means (SDs). ^a,b,c^ Superscripts reflect significant differences between and within the three study samples for the respective variables based on Welch’s t tests, where a > b > c; groups with the same superscript(s) do not differ significantly from one another. AUDIT, BD, age, and recall scores had a somewhat positive skew.

#### Cognitive tasks

##### Digit-span

Participants completed the forward digit-span task requiring them to repeat verbally presented sequences of numbers of progressive length (3–9 digits). This task served as a distraction to minimize expectation that memory would also be assessed for the sentence task.

##### Self-referent sentences task

Participants completed an incidental encoding task. Participants were asked whether they felt 30 short sentences (e.g., “I am rational”) described them (“yes” or “no”) to determine self-reference. Sentences contained 10 positive, 10 neutral, and 10 negative adjectives from the English Word Database of Emotional Terms (EMOTE; Gruhn, 2016), presented in a random order. The items were selected from the EMOTE database based on the following ranges (on a scale of 1–7): negative 1–2.5, neutral 3–5, and positive 5.5–7. Moreover, items within each valence category were matched for numbers of letters and syllables, word frequency, imagery, concreteness, meaning, and familiarity (ps > 0.05). To further validate the emotional dimensions in our sample, participants rated the valence and arousal of each sentence after the memory task on the second day. Negative sentences were rated as more negative in valence than neutral sentences, d = −2.05, *p* < 0.001, and positive sentences were rated as more positive in valence than neutral sentences, d = 1.50, *p* < 0.001. There were no significant differences in arousal ratings, *F*(2, 112) = 1.23, *p* = 0.30, η_G_^2^ = 0.02, supporting that an NMB would be primarily driven by valence.

##### Free recall

Approximately 24 h after the incidental encoding of sentences, we asked participants to type out as many sentences as they could recall from the sentences task within 2 min. Memory bias scores were calculated by subtracting correctly recalled negative sentences from correctly recalled positive sentences, such that negative bias scores reflect an NMB and positive scores reflect a positive memory bias. Bias scores were further defined according to whether the respective sentences had previously been deemed self-referent or not by each participant on Day 1.

#### Questionnaires

##### Alcohol use disorders identification test (AUDIT)

The AUDIT (Saunders et al., 1993) consists of 10 items rated on a 5-point scale (0–4) that assesses the frequency and amount of alcohol consumption and alcohol-related problems (e.g., feeling guilty after drinking) in the past year. A score of 8 or more indicates risky drinking habits. The AUDIT has been validated across genders and various ethnicities (Saunders et al., 1993; World Health Organization, 2001) and has demonstrated good internal consistency (Cronbach’s α = 0.83; Selin, 2003).

##### Timeline followback method- alcohol (TLFB)

Participants reported estimates of their daily alcohol consumption during a 60-day TLFB (Sobell and Sobell, 1995) interview to measure the number of BD (5 standard drinks in males, 4 in females) episodes. The TLFB has been validated in males and females over the age of 14 in clinical and nonclinical samples (Sobell and Sobell, 1995) and demonstrates high test-retest reliability for BD episodes (ICC = 0.79; Cohen and Vinson, 1995).

##### Drinking motives questionnaire-revised (DMQ-R)

The DMQ-R (Cooper, 1994) contains 20 statements representing four categories of drinking motivations, including coping, enhancement, social, and conformity. Here, we focus on the coping subscale to address our *a priori* hypotheses. Motivations are rated on a 5-point scale based on how frequently they occur (Never/Almost Never to Always/Almost Always). The DMQ-R was validated in samples of adolescents and adults (Cooper et al., 1992; Cooper, 1994), its subscale structure holds across gender, ethnicities, and age, and it shows good internal consistency (Cronbach’s α = 0.84; Harbke et al., 2019).

##### The depression anxiety stress scale (DASS-21)

The DASS-21 (Lovibond and Lovibond, 1995) measures self-reported symptoms of depression, anxiety, and stress symptoms experienced in the past week. Each subscale contains seven items rated on a 4-point scale. Scores range from 0 to 42 with greater numbers indicating greater severity. This scale shows good internal consistency (Cronbach’s α = 0.83–0.85), as well as good construct validity across ethnicities (Norton, 2007) and in clinical and nonclinical samples (Osman et al., 2012).

##### Bidimensional impression management index (BIMI)

Social desirability was assessed using the BIMI (Blasberg et al., 2013), which distinguishes between agentic (exaggeration of social status or intellect) and communal impression management (denying socially deviant impulses and exaggerating virtuous attributes). The mean score on the agentic subscale in our sample exactly equals the norm from Blasberg et al. (2013) for honest responses (Welch’s t d = 0, *p* = 1.0), and is significantly lower (i.e., more honest) than the norms for ‘faking good’, d = −0.92, *p* < 0.001. Scores on the communal subscale from our sample were significantly greater than the norms for honest, d = 0.33, *p* = 0.05, but were also significantly lower than norms for ‘faking good’, d = −0.38, *p* = 0.01. These patterns together with the fact that this study was not looking at prosocial behavior support an honest profile of responses by the current sample.

#### Statistical analyses

As some alcohol consumption and recall variables were positively skewed, we assessed bivariate correlations using Spearman’s rho among the variables of interest: Number of BD episodes, AUDIT, coping, depression, bias scores, and total recall. Based on these relations and our hypotheses, we used PROCESS v3.5 in R (Model 6; Hayes, 2017) to assess serial indirect relations between alcohol use and an NMB through depression and coping. Predictor, mediator, and outcome variables were converted to standardized scores. Significance of indirect effects is interpreted relative to 95% confidence intervals (CIs) constructed with 5,000 bootstrap samples with a consistent randomly selected seed (31216) across analyses; *p* values are reported for direct and total effects. Regression assumptions were satisfied for all models.

### Results

#### Memory performance

Overall recall ranged from 0 to 5 sentences (see Table 1 for descriptive statistics). Using Wilcoxon signed-rank test, there was no significant difference in overall recall for negative and positive sentences (*p* = 0.89). However, for sentences deemed self-referent there were significantly fewer negative than positive sentences recalled (*p* < 0.01); the reverse was true for sentences rated not self (p < 0.01). Self-bias scores ranged from −2 to 2 and not-self bias from −3 to 2. Thus, despite an overall positive bias, there was variation across individuals in demonstrating an NMB or positive memory bias.

#### Correlations and regressions

As seen in Table 2, greater scores on the AUDIT were significantly related to lower self-bias scores, consistent with a self-NMB. BD episodes, however, were not significantly related to a self-bias score. Higher AUDIT and more BD episodes were both significantly correlated with endorsement of coping motivations; AUDIT was also highly positively correlated with depressive symptoms whereas BD failed to reach significance. Moreover, greater depression and endorsement of coping motivations significantly predicted lower bias scores only for self-referent sentences. In contrast, bias scores for sentences deemed “not-self” only revealed very small (rhos <0.15) and nonsignificant relations with variables of interest. Likewise, total recall performance was not significantly related to BD, AUDIT, depression, or coping motivation scores. The above results indicate that a tendency toward an NMB in individuals high on the aforementioned variables primarily occurs for information deemed self-referent.

**Table 2:** Correlation matrix among the alcohol, clinical, and memory variables of interest in Study 1.

	**AUDIT**	**BD episodes**	**Depression**	**Coping**
**Depression**	0.46***	0.05		
**Coping**	0.65***	0.37**	0.51***	
**Self-Bias**	−0.38**	−0.10	−0.46***	−0.27*
**Not-self ** **NMB**	0.05	0.03	0.14	0.09
**Total recall**	−0.18	−0.16	0.12	−0.13

AUDIT, Alcohol Use Disorders Identification Test; BD, Binge Drinking.

Correlation coefficients are reported as Spearman’s rho. Bold values are significant relations between main variables of interest.

*p < 0.05.

*p < 0.01 Self-Bias = self-positive–self-negative recalled.

***p < 0.001 Not-self Bias = not-self positive–not-self negative recalled.

To better understand the observed relations of AUDIT and depression with the self-bias scores, we used multiple regression to assess the relative contributions of recall for self-referent negative and positive sentences. Together they explain 8% of the variance in AUDIT (R^2^ = 0.08, *p* = 0.05) and 13% of the variance in depression scores (R^2^ = 0.13, p < 0.01). In both cases, higher scores on these measures were associated with a pattern of recalling more negative (β = 0.21, β = 0.36, respectively) and fewer positive self-referent sentences (β = −0.26, β = −0.19, respectively).

#### Serial multiple mediation through coping and depression

Given Carbia et al.’s (2020) findings of the association between BD and an NMB, we first assessed the serial mediation model on self-bias scores with number of BD episodes as the predictor; the current lack of bivariate relation does not preclude the presence of meaningful indirect effects (Hayes, 2017). With BD as the predictor, the specific total indirect effect was significant such that a greater frequency of BD days predicted a tendency to endorse coping motives, which predicted greater depressive symptoms, and, in turn, lower self-bias scores, β = −0.12, [−0.28, −0.01]. An increase of 1 SD or approximately 4 BD episodes in the 60-day period corresponded with a 12% lower self-bias score through endorsement of coping motivations and depression. The direct effect of BD on self-bias memory scores did not explain significant unique variance (*p* = 0.61).

**Figure 1:**
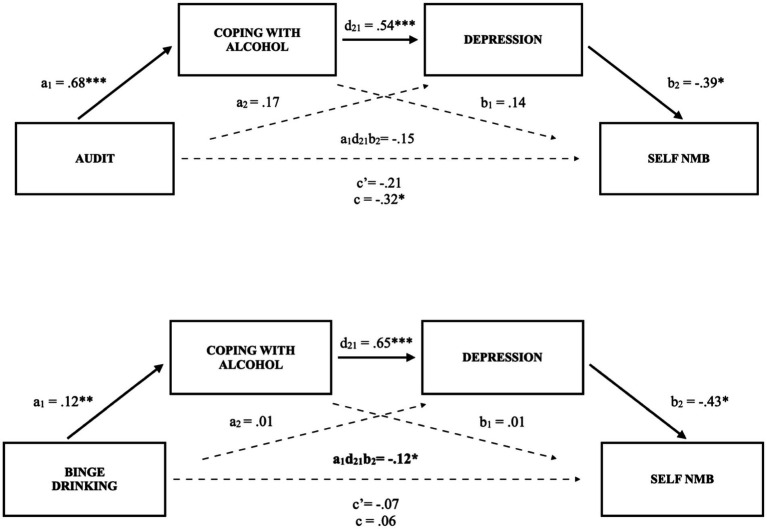
Serial multiple mediation model of the relations between problematic drinking (AUDIT scores) and binge drinking with negative self-referent memory bias (self-NMB) through coping motivations and depressive symptoms in undergraduate female students. (Study 1). Solid lines and bolded parameters reflect significant relations: *p < 0.05, **p < 0.01, ***p < 0.001.

The overall relation (total effect) between AUDIT and self-bias memory scores was significant (*p* = 0.02), as were the individual components of the indirect pathway, supporting the predictive value of AUDIT scores on coping motivations, of coping motivations on depressive symptoms, and of depressive symptoms on the self-bias scores (see Figure 1). The specific total indirect effect (i.e., serial path), however, just failed to reach significance, β = −0.15 [−0.33, 0.003]. The direct effect of AUDIT on a self-NMB was not significant (*p* = 0.22), supporting that the relation between alcohol use and a self-NMB is primarily explained *via* coping motivations and depressive symptoms.

#### Model validation

Although our cross-sectional design cannot support causal inference, the mediation verbiage represents the underlying theory. That is, the theoretical basis behind the above models is that excessive alcohol use (either problematic drinking or BD) predicts lower self-bias scores through greater coping motivations, which predicts greater depression. To test the above models, we examined alternatives by reordering the predictor and the intervening variables. In the BD model, swapping the order of mediator variables resulted in no significant pathways, whereas in the AUDIT model, only the specific indirect effect through depression was significant, β = −0.22 [−0.47, −0.002]. With depression as the predictor in both AUDIT and BD models, the direct effects were significant (p = 0.02; *p* = 0.01) whereas none of the indirect pathways were significant. Comparatively, with coping motivations as the predictor in the AUDIT model, neither direct nor indirect effects were significant. With coping motivations as the predictor in the BD model, only the specific indirect pathway through depression was significant, β = −0.27, [−0.51, −0.03]. Additionally, the other DMQ-R subscales of enhancement, social, and conformity motivations failed to show significant indirect effects when controlling for coping motivations. Likewise, anxiety and stress subscales of the DASS-21 failed to show significant indirect effects when controlling for depression. These findings further support that problematic drinking and BD are primarily related to lower self-bias memory scores through the respective indirect effects of depression and coping motivations in the predicted manner.

### Discussion

As expected, problematic drinking, coping, and depression scores were particularly related to a tendency to preferentially recall self-referent negative information, as opposed to sentences that participants deemed not self-referent. This aligns with previous findings that the NMB tends to be stronger for self-referent stimuli in individuals with depression (Zupan et al., 2017). Moreover, an indirect path through coping motivations and depression explained the relations between BD and problematic drinking with lower memory bias scores for self-referent sentences.

Notably, the relation between alcohol use and an NMB has so far only been found in females (Carbia et al., 2020). Study 1 built upon this relation in assessing the mediating role of coping motivations and depression in females. Sex and gender are important, although somewhat complex, considerations in the discussion of alcohol use and emotional dysregulation. Sex refers to a biological trait that influences the development of the brain and body (Sanchis-Segura and Becker, 2016) and contributes to how individuals interact with their environment. Gender refers to individual embodiments or expressions that interact with but may not align with sex (Sanchis-Segura and Becker, 2016). Gendered environmental experiences may subsequently influence the brain and alter behavior (Sanchis-Segura and Becker, 2016). For example, experiences of sexual trauma are significantly more common in women (Olff, 2017), which may dysregulate brain functioning and emotional responses and result in greater use of alcohol to cope with related symptoms (Ullman et al., 2013; Hammerslag and Gulley, 2016). Thus, both sex and gender involve biological and environmental interactions and play a key role in vulnerabilities and responses to substance use. These factors warrant attention in relation to self-NMB.

## Study 2

Study 2 provides a replication of the mediating roles of coping motivations and depression in the relation between alcohol use and an NMB. Given that Carbia et al. (2020) only observed significant NMB relations with BD in women, we also investigate whether sex/gender moderates the above indirect relations. Of note, our current aims were not to disentangle sex and gender effects, and as detailed below these variables were largely redundant in our study; thus, we refer to sex only throughout. Further, as outlined below, given low overall recall in Study 1, we modified the memory paradigm to enhance task sensitivity.

### Methods

#### Participants

Using the coefficients obtained from the model with AUDIT scores as the predictor, which just failed to reach significance in Study 1 (see Figure 1), we applied Schoemann et al.’s (2017) application that estimated a target sample size of n = 70 for a serial mediation effect with 80% power. Thus, we aimed to recruit a minimum of 70 females and 70 males to target serial mediation within each sex. Across two terms, we recruited 138 female and 76 male students *via* the same undergraduate pool as for Study 1. There was limited discrepancy between sex and gender identifications (i.e., 98% of females identified as women and 97% of males identified as men). Thus, as noted, analyses were conducted by sex; however, results may be indicative of either sex-based (i.e., biological) differences or gendered (i.e., environmental) experiences.

In contrast to Study 1 where 7% of students reported abstaining from alcohol (i.e., a score of 0 on the AUDIT), this percentage was almost five times higher in the Study 2 sample (33%). As the reasons for abstinence may vary, this high proportion of the sample reporting no use may impede clear interpretation of the relations with problematic alcohol use. To address this concern, we analyzed the data with and without those who reported no consumption; the ratio of males:females across the original and alcohol-using samples was similar (0.55; 0.53, respectively; see Table 1). The pattern of findings was similar across these analyses in terms of the strength of relations and their interpretation relative to significance decision thresholds. We report the findings from the smaller sample to better represent the relations with problematic drinking (AUDIT scores).

#### Materials and procedure

Questionnaires administered were the same as in Study 1 along with the inclusion of the Personal Attributes Questionnaire (PAQ; Spence and Helmreich, 1978) to assess individuals’ alignment with gender roles; descriptive statistics are reported in Table 1. AUDIT showed good internal consistency in the Study 2 sample (Cronbach’s α = 0.85), the depression subscale of the DASS-21 showed excellent internal consistency (α = 0.90), and the coping motivations subscale of the DMQ-R showed acceptable internal consistency (α = 0.77). To enhance overall recall and sensitivity of the bias scores, however, we made a number of modifications to the main memory task in this iteration of the study. First, we increased the focus on the positive and negative stimuli of interest, and presented 14 negative and 14 positive sentences in a random order. Only 2 neutral sentences were included at the beginning and end of the Self-Referent Sentences Task to control for primacy and recency effects. In addition, we included a second task that asked participants to identify the valence (“positive” or “negative”) of the adjectives from the Self-Referent Sentence Task in order to re-expose them to the key stimuli. On Day 2, participants were asked to recall as many sentences as they could, with one of the neutral sentences used as a reminder of the task (e.g., “I am rational”).

In contrast to the direct interaction and interview style used in Study 1, we also moved Study 2 to an anonymous online survey format. More specifically, participants completed the study remotely *via* Qualtrics^1^ and Pavlovia^2^ online platforms. Results of the BIMI agentic and community subscales were significantly lower than the student norms for ‘faking good’ (d = −1.60, *p* < 0.001; d = −0.56, p < 0.001), indicating honest response styles.

### Statistical analyses

Following bivariate correlation and regression analyses as for Study 1, we conducted a conditional process analysis *via* PROCESS v3.5 (Model 92; Hayes, 2017) to assess the serial mediation models by sex. We also explored bivariate relations with femininity and masculinity. Regression assumptions were satisfied for all models.

### Results

#### Memory performance

Recall ranged from 0 to 14 sentences (M = 3.4, SD = 2.6), indicating that alterations to the paradigm were successful in raising overall recall levels compared to Study 1, Welch’s t (168) = 5.38, *p* < 0.001. In both sexes, there were significantly fewer self-negative sentences recalled relative to self-positive (Wilcoxon signed-rank test; female, p < 0.001; male, p < 0.001). For sentences rated “not-self,” there were more negative relative to positive sentences for females, *p* < 0.01, whereas there was no significant difference for males, *p* = 0.18. Notably, there was variation in memory biases across individuals as self-bias and not-self bias scores ranged from-4 to 5 and-5 to 5 in females, respectively, and from −2 to 3 and −5 to 4 in males, respectively.

#### Correlations and regressions

Replicating the pattern of results from Study 1, the relations between AUDIT, coping motivations, and depression with lower self-referent memory bias scores were of similar magnitude among females in Study 2; moreover, all three relations were significant (see Table 3). In males, depressive symptoms were significantly related to lower self-referent bias scores. Further, more BD episodes revealed small-medium relations to lower self-referent bias scores, but failed to reach significance, whereas AUDIT and coping motivations did not relate to bias scores in males (Table 3).

**Table 3:** Correlation matrix among female (below diagonal) and male (above) student alcohol, clinical, and memory variables of interest in Study 2.

	AUDIT	BD days	Depression	Coping	Self-Bias	Not-Self Bias	**Total Recall**
**AUDIT**		0.51***	0.26*	0.58***	−0.04	0.25	0.02
**BD days**	0.60***		0.08	0.23	0.23	−0.08	0.13
**Depression**	0.21*	0.04		0.37**	−0.27*	0.34	0.09
**Coping**	0.46***	0.23**	0.47***		0.04	0.28	<0.01
**Self-Bias**	−0.25*	−0.16	−0.38**	−0.27**		−0.40	−0.40
**Not-Self Bias**	0.11	<0.01	−0.33*	0.23	−0.38***		0.12
**Total Recall**	0.08	0.13	0.05	0.05	0.35*	0.24	

AUDIT, Alcohol Use Disorders Identification Test; BD, Binge Drinking.

Correlation coefficients are reported as Spearman’s rho. Bold values are significant relations between main variables of interest.

*p < 0.05.

**p < 0.01 Self-Bias = self-positive– self-negative recalled.

***p < 0.001 Not-self Bias = not-self positive–not-self negative recalled.

Regression models were fit to examine the unique relations of self-positive and self-negative sentences recalled to the above three variables for females and the two small-medium relations for males. In females, higher AUDIT, coping, and depression scores were associated with significantly greater recall of negative self-referent sentences (β = 0.17, *p* < 0.05, β = 0.26, p < 0.01, β = 0.38, p < 0.001, respectively); depression was also associated with significantly lower recall of positive self-referent sentences (β = −0.28, p < 0.01). Comparatively, only depression was significantly related to greater recall of negative self-referent sentences in males (β = 0.34, *p* = 0.02).

To explore potential relations with gender, and specifically alignment with gender roles, we examined the relations of femininity and masculinity scores with the five key variables of interest (self-bias, AUDIT, BD, coping, depression). Among females, femininity was moderately correlated with higher AUDIT scores rho = 0.25, p < 0.05, and more BD episodes, rho = 0.29, p < 0.01, whereas masculinity related to more positive self-bias memory, rho = 0.28, p < 0.01, and lower depression, rho = −0.47, *p* < 0.001. There were no significant relations among these measures for males.

#### Moderated serial multiple mediation through coping and depression

**Figure 2:**
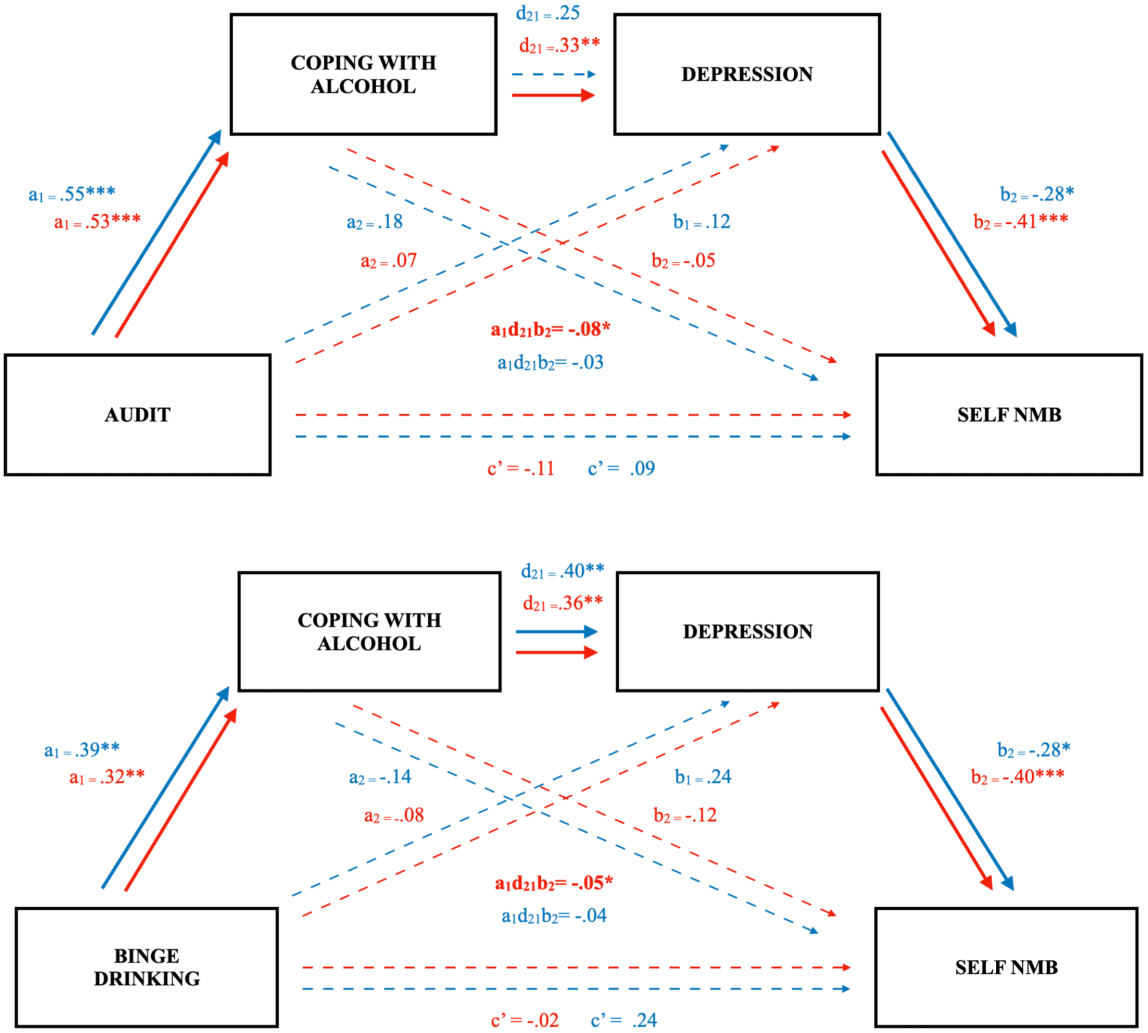
Indirect effects of coping motivations and depression on the relation between problematic drinking, binge drinking, and a self-referent memory bias in undergraduate females, but not males (Study 2). Figure depicts separate serial mediation models with females in red, males in blue. Solid lines and bolded parameters reflect significant relations: *p < 0.05, **p < 0.01, ***p < 0.001.

Supporting assertions on the same model in Study 1, we found that in females the specific total indirect (i.e., serial) pathway was significant such that more problematic alcohol use predicted greater coping motivations, which predicted greater depressive symptoms, which predicted a lower self-bias score, β = −0.08, [−0.19, −0.02] (see Figure 2). That is a 1 SD greater AUDIT score predicted an 8.3% lower self-referent bias score, through endorsement of coping motivations and greater depressive symptoms. The same was not true for male students with AUDIT scores, for whom the specific total indirect effect failed to reach significance, β = −0.03, [−0.10, 0.01]. Despite the sex difference in significance of the indirect pathway, the index of moderated mediation failed to reach significance, β = −0.05, [−0.20, 0.05]. After accounting for coping motivations and depression, the direct effects of AUDIT on self-bias scores were also nonsignificant in females (*p* = 0.43) and males (*p* = 0.51).

In the model with BD as the predictor, the specific total indirect pathway was again significant for females, such that a greater number of BD days was associated with greater coping motivations, which predicted greater depressive symptoms, which predicted a lower self-bias score, β = −0.05, [−0.12, −0.004], consistent with Study 1. That is 1 SD greater BD episodes (~ 4 more episodes) predicted a 4.9% lower self-bias score. Comparatively, the serial indirect pathway for males was of a similar magnitude, but again not significant, β = −0.04 [−0.11, 0.01]. The index of moderated mediation was also not significant, β = −0.01 [−0.10, 0.08]. As expected, the direct paths failed to account for unique variance in self-bias memory scores (females, *p* = 0.86; males, *p* = 0.18). Notably, in both AUDIT and BD models, the separate specific indirect pathways through only depression or only coping were not significant (see Figure 2).

### Discussion

In sum, with both problematic alcohol use and BD episodes as predictors, we replicated a significant indirect relation with more negative self-referent memory biases as explained through coping motivations and depression among females. Although the pattern was similar and relations were of similar magnitude for males, these associations failed to reach significance.

## Study 3

We conducted Studies 1 and 2 on undergraduate student samples given characteristically elevated rates of alcohol consumption and depressive symptoms in this population (Geisner et al., 2012). Here, we expand our investigation to a community sample to assess the generalizability of proposed models.

### Methods

Based on *a priori* power analyses detailed in Study 2, we recruited 165 Canadian community participants *via* the Honeybee Hub recruitment platform^3^ for a $10 incentive ($5/day). Due to incomplete or invalid survey responses, 44 male and 27 female responses were excluded, leaving 47 male and 48 female participants. Only 9.4% of the remaining sample reported no alcohol consumption on the AUDIT. Sample characteristics are summarized in Table 1. The procedures and measures were otherwise identical to Study 2. The AUDIT showed good internal consistency in the Study 3 sample (Cronbach’s α = 0.89), as did the depression subscale of the DASS-21 (α = 0.87), and the coping motivations subscale of the DMQ-R (α = 0.89). Scores on the BIMI were significantly lower than the norms reported for the online community sample in Blasberg et al. (2013) for “faking good” on both the agentic, d = −2.39, *p* < 0.001, and community subscales, d = −3.23, p < 0.001, indicating honest response styles. Regression assumptions were satisfied for all models.

### Results

#### Memory performance

Recall ranged from 0 to 11 sentences (M = 3.2, SD = 2.5). In females and males, there were significantly fewer self-negative sentences recalled relative to self-positive (Wilcoxon signed-rank test, both p < 0.001) and, conversely, more not-self negative sentences relative to not-self positive (females *p* = 0.01; males p < 0.001). In females, self-bias and not-self bias scores ranged from −1 to 7 and −3 to 2, and in males from −1 to 8 and −4 to 1, respectively.

#### Correlations and regressions

As seen in Table 4, higher scores on the AUDIT, depressive symptoms, and coping motivations in males revealed medium-sized and significant relations to a greater NMB for self-referent sentences. Further, males showed small-medium relations between BD days with a negative self-memory bias, though these failed to reach significance. Unlike Studies 1 and 2, among females from the community, only greater endorsement of coping motivations revealed a small-medium, but nonsignificant relation to lower self-bias scores. Notably, in females, greater alignment with feminine gender roles was moderately related to lower depression, but failed to reach significance (rho = −0.24, *p* = 0.09). Masculinity and femininity were not related to variables of interest in males.

**Table 4:** Correlation matrix for females (below diagonal) and males (above) in the community sample for alcohol, clinical, and memory variables of interest in Study 3.

	**AUDIT**	**BD days**	**Depression**	**Coping**	**Self-Bias**	**Not-SelfBias**	**Total Recall**
**AUDIT**		0.63***	0.26*	0.72***	−0.32*	−0.16	0.11
**BD days**	0.74***		0.39**	0.45***	−0.27	0.18	0.10
**Depression**	0.10	0.13		−0.02	−0.14*	0.04	0.02
**Coping**	0.70***	0.53***	0.27*		−0.33*	0.04	0.26
**Self-Bias**	0.06	0.06	−0.13	−0.27		0.24	0.72*
**Not-Self Bias**	−0.18	0.23	0.13	0.28	−0.45***		0.55*
**Total Recall**	−0.05	−0.01	0.17	0.10	0.68*	−0.52*	

AUDIT, Alcohol Use Disorders Identification Test; BD, Binge Drinking.

Correlation coefficients are reported as Spearman’s rho. Bold values are significant relations between main variables of interest.

*p < 0.05.

**p < 0.01 Self-Bias = self-positive–self-negative recalled.

***p < 0.001 Not-self Bias = not-self positive–not-self negative recalled.

Nonetheless, depression scores among females predicted significantly greater recall of negative self-referent sentences (β = 0.35, *p* = 0.02) through regression analyses. Similarly, depression in males was also associated with significantly greater recall of negative self-referent sentences (β = 0.29, *p* = 0.04), while coping motives were related to significantly lower recall of self-referent positive sentences (β = −0.39, p < 0.001).

#### Moderated serial multiple mediation through coping and depression

**Figure 3:**
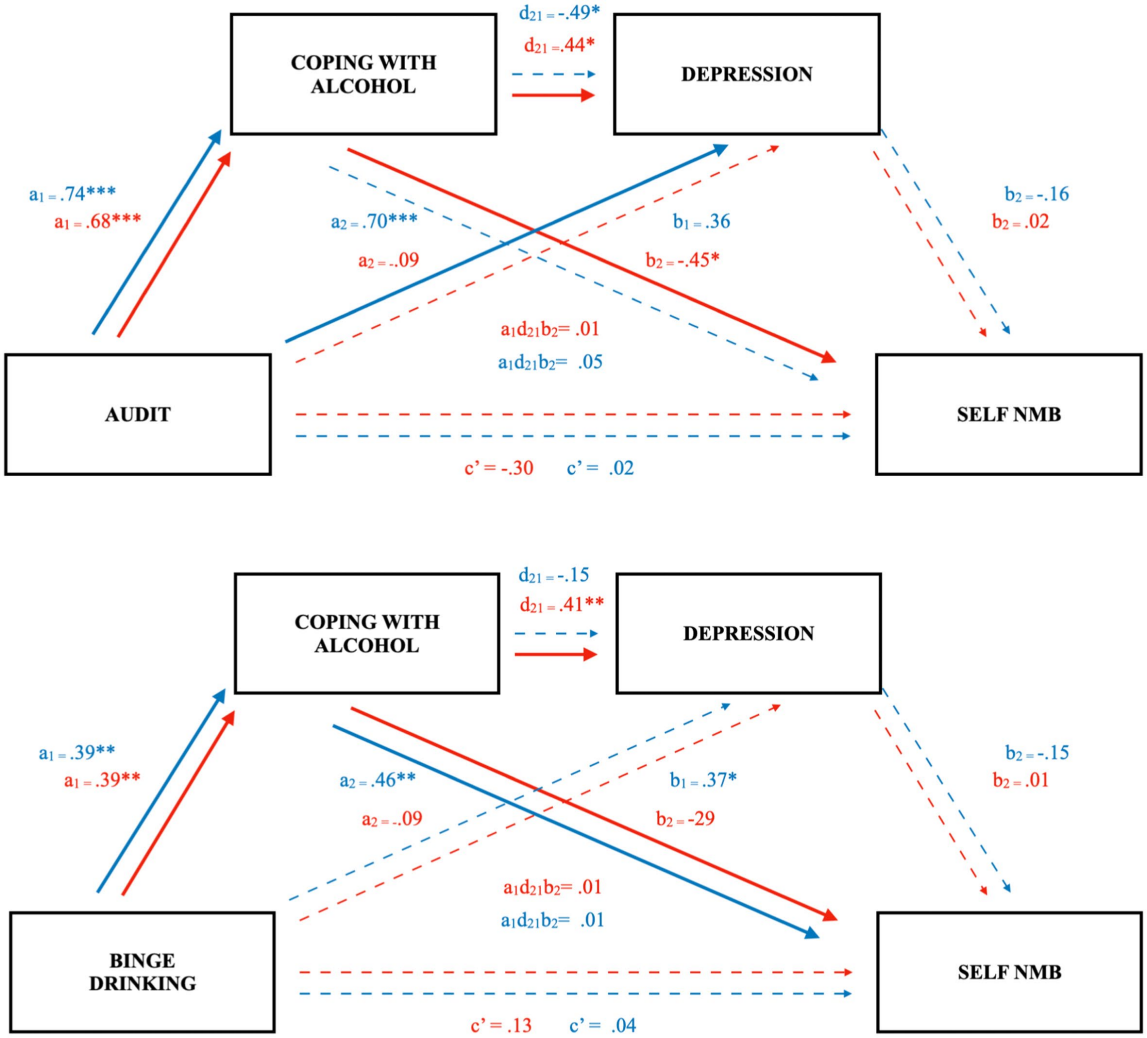
Coping mediates the relation between problematic drinking and a self-referent negative emotional memory bias in community females but not males (Study 3). Figure depicts separate serial mediation models with females in red, males in blue. Solid lines and bolded parameters reflect significant relations: *p < 0.05, **p < 0.01, ***p < 0.001.

As in Study 2, we assessed the moderated serial multiple mediation models in the community sample. In contrast to the undergraduate samples, the paths from depression to self-bias scores were nonsignificant, precluding the total specific indirect paths through both coping and depression (see Figure 3).

Notably, however, with BD as the predictor, the specific indirect pathway through coping was significant in both females, β = −0.09, [−0.39, −0.01] and males, β = −0.24, [−0.61, −0.05]. As such, 1 SD greater BD episodes (2 episodes for females and 3 for males) predicted 9 and 24% lower self-bias scores, respectively (see Figure 3). Again, the index of moderated mediation was not significant, β = 0.15, [−0.18, 0.52]. Finally, the direct effect between BD and self-referent memory bias was not significant in either females (*p* = 0.37) or males (*p* = 0.81).

For females, the specific indirect effect for AUDIT through coping was significant (β = −0.43, [−0.79, −0.13]; see Figure 3), such that 1 SD increase in AUDIT scores predicted a 43% lower self-bias score through coping motivations in community females. In males, this relation was half the magnitude and not significant, β = −0.22, [−0.48, 0.07]. Despite discrepancy in path coefficients between the sexes, the index of moderated mediation was not significant, β = −0.21, [−0.67, 0.17]. The direct effect for AUDIT was not significant for females (*p* = 0.10) or males (*p* = 0.91).

As males and females in the community sample were significantly older than students in Studies 1 and 2 (Table 1), we assessed the relations between age and bias scores; however, it was not significant (rho = 0.27, *p* = 0.07).

To partially address modest sample size limitations in the above three studies, we ran omnibus conditional process analyses pooling the data from all participants (N = 292); results revealed a significant index of moderated mediation, supporting a difference in relations among problematic alcohol use (AUDIT), depression, endorsement of coping motivations, and a self-bias score in females relative to males β = −0.07, [−0.14, −0.02]. Indeed, the full mediation model was significant in females β = −0.07, [−0.12, −0.02], but not for males, β = 0.003, [−0.03, 0.04]. Similarly, results for the BD model supported the full mediation model in females β = −0.05, [−0.10, −0.02] and not males, β = −0.01, [−0.03, 0.01], although the index of moderated mediation failed to reach significance, β = −0.04, [−0.09, 0.001].

### Discussion

We found an indirect effect of endorsement of coping motivations on the relation between alcohol consumption variables (both AUDIT scores and BD episodes) and an NMB in community females. Interestingly, we also found that endorsement of coping motivations was a significant intervening variable for only the BD model in community males, in contrast to Study 2 findings. Study 3 supports the relevance of maladaptive behaviors linked with emotional dysregulation (i.e., drinking to cope with negative affect), in the proposed relation between excessive alcohol use and preferential recall of self-referent negative stimuli. When pooling all three studies, we also found support for the full indirect effect in AUDIT and BD models in females only, and moderated mediation in the AUDIT model supported a significant difference between females and males.

## General discussion

In this three-part study, we examined whether coping motivations and depressive symptoms serially explain the relationship between problematic drinking and BD with a self-referent NMB in undergraduate and community samples, as well as the moderating role of sex. Overall, results generally replicated across studies with minor nuances in findings. Across all three samples, we found bivariate relations between a self-referent NMB and problematic drinking (AUDIT scores), depressive symptoms, and endorsement of coping motives. Further, across both student samples, multiple linear regression models found that greater recall of self-referent negative sentences was associated with higher levels of problematic drinking and depressive symptoms, and that lower recall of self-referent positive sentences associated with depressive symptoms. More central to our study aims, findings from both student samples supported the serial mediation model with an indirect pathway through coping motivations and depressive symptoms among females for both BD and problematic drinking, whereas this path was weaker for males and nonsignificant. Comparatively, in the community sample, the proposed model was partially replicated as only the specific indirect pathways through coping motivations were significant for both sexes with BD episodes and in females with AUDIT scores.

### The role of sex in alcohol consumption behaviors and consequences

In female undergraduate students, excessive alcohol use patterns *via* the AUDIT consistently related to a greater tendency to recall self-referent negative stimuli. Excessive alcohol use is purported to give rise to an NMB related to lower prefrontal-cortex inhibition of the amygdala (Stephens and Duka, 2008). In the current study, the more robust pattern of findings in undergraduate females relative to males may relate to different neural vulnerabilities and responses to alcohol consumption. For example, high levels of sex-steroid receptor expression in the prefrontal cortex and amygdala relate to sexually dimorphic pubertal development and functionality of these regions, resulting in a greater impact of alcohol consumption on dysregulation of emotions in females, relative to males (Hammerslag and Gulley, 2016). Thus, female students who excessively consume alcohol during university, a formative developmental period, may be particularly vulnerable to alcohol-induced neurological changes in regions associated with processing emotional memories (e.g., hippocampus, amygdala, and prefrontal cortex; Stephens and Duka, 2008). Further, emotional dysregulation associated with excessive alcohol consumption may manifest in the form of experiencing depressive symptoms and turning to maladaptive coping mechanisms for negative emotions (Koob, 2013).

Comparatively, problematic alcohol use among male participants in the community sample was significantly correlated with the self-NMB, whereas this was not the case for female participants. Though somewhat unexpected based on findings of the student samples in Study 1 and 2, along with previous research (Carbia et al., 2020), this may be explained by the higher rates of alcohol consumption observed in community males (nearly double their female counterparts, as well as both male and female students). Thus, whereas certain vulnerabilities, whether neural or behaviorally based, may render females more sensitive to an NMB at lower levels of consumption, more severe patterns of problematic drinking may still contribute to emergence of an NMB in males. Further, although there was no longer a bivariate relationship for females, alcohol use remained a predictor of a self-NMB *via* endorsement of coping motivations. Future research will be useful for exploring potential dose-dependent relationships across sexes and other demographics (e.g., age and student status).

### Serial mediation through coping motivations and depressive symptoms

As hypothesized, across all models, alcohol variables did not reveal a direct effect on the NMB after accounting for coping motivations and depression. Rather, we observed a significant intervening effect of coping motivations in females whether independently (Figure 3) or as part of a serial mediation with depressive symptoms (Figures 1, 2). Individuals who are motivated to consume alcohol to cope with negative affect may be more likely to experience an NMB *via* reinforcement of alcohol cravings in response to negative affective cues, greater cognitive and emotional dysregulation, and higher levels of alcohol consumption (Zack et al., 1999, 2002; Stephens and Duka, 2008; Foster et al., 2014; Decaluwe et al., 2019). In fact, the serial models in Studies 1 and 2 for undergraduate females support that excessive use of alcohol may influence an individual to use it as a coping mechanism through negative reinforcement (Koob, 2013) and that this may precipitate onset or worsening of depressive symptoms (Connell et al., 2015), further contributing to the self-referent NMB (Disner et al., 2011).

Interestingly, depression was not supported as a mediator in Study 3. The discrepancies among undergraduate and community female samples may in part be explained by differences in prevalence of depressive symptoms, such that Study 1 students had the highest levels, followed by Study 2 students, and community members reporting the lowest. Additionally, whereas relations between alcohol measures and coping were robust and remarkably similar between sexes for all samples (Tables 3, 4; Figures 2, 3), the relations with depression were more discrepant and its relations with the NMB were relatively weak in the community sample. Furthermore, the community sample was on average older than the student samples, which is potentially indicative of a protective effect of age (Reed and Carstensen, 2012). Indeed, previous studies have also found that the NMB is especially pronounced in younger depressed participants and decreases with age (Zupan et al., 2017). Thus, relations between alcohol use and the NMB may be less reliant on depressive symptoms with age, whereas the full serial model appears especially relevant for adolescents and young adults.

### The role of sex in the serial mediation model

Research on sex/gender differences in the tendency to endorse coping motivations is mixed, with adolescent girls between the ages of 13–19 years reporting greater coping motives than boys, whereas no gender differences are seen in college-aged students (for review see Kuntsche et al., 2006). In Study 3, we observed that coping motivations explained the relation between BD and an NMB in both males and females. Further, in Study 3, the male community sample endorsed significantly higher rates of coping motivations relative to male undergraduates. As such, in tandem with elevated alcohol consumption levels in the community males, cognitive emotional biases related to higher rates of maladaptive coping that are thought to underlie the NMB may explain the significant effect of coping motivations in this group. Interestingly, we found that in both male samples, coping motivations were not significantly related to depressive symptoms in the serial mediation models, whereas this relationship was significant for all female samples. This corresponds with previous findings that in females, endorsement of coping motivations was significantly related to higher depressive symptoms, whereas this relationship was not significant in males (Foster et al., 2014).

Sex and/or gender can play a role in the prevalence of and response to depression. For example, a significant increase in depression is seen in only females during Tanner stage III in mid-puberty (Hammerslag and Gulley, 2016). Moreover, socialization and gender inequalities may contribute to negative self-referent biases and lower self-esteem in vulnerable individuals (van der Aar et al., 2018; Bone et al., 2021). To elaborate, gender-informed cognitive models of depression suggest that adolescent girls are more vulnerable to depression due to differences in rumination and negative inferential style, a greater likelihood of experiencing negative life events, and a greater tendency to develop depressive symptoms in response to such events (Hankin and Abramson, 2001). This coincides with the finding that in undergraduate females, alignment with feminine gender roles was related to greater alcohol consumption whereas greater endorsement of masculine gender roles was associated with lower depression scores. Conversely, in males, gender role alignment did not relate to variables of interest, which may suggest experiences of gender and gender roles play a bigger part for females with respect to alcohol consumption, depression, and potentially cognitive sequelae, while this does not appear true for males. Surprisingly, this contradicts previous research that found that alignment with feminine roles assessed by the same measure was negatively related to alcohol consumption (Peralta et al., 2010). However, Peralta et al. (2010) did not disaggregate findings by sex identification, possibly obscuring differences. This may also be reflective of differences in socio-cultural context by time or location of samples.

It is important, however, not to overstate the immutability of sex/gender differences. While literature shows females are likely to experience depression and have a greater tendency toward an NMB than males (Hammerslag and Gulley, 2016; Dir et al., 2017; Hardee et al., 2017), males who are depressed may also show differential responses relative to males who are not depressed. That is, while there may be differences between males and females in general, the differences between males and females with depression may be less striking. Indeed, in both undergraduate and community males and females, depressive symptoms predicted greater recall of self-negative sentences, indicating a common cognitive risk. Further, the pathway from depressive symptoms to the NMB was significant in both female and male students. As such, future research may investigate these relations in a sample of males with diagnosed depressive disorders, who may be more likely to present with negatively biased cognition.

### Strengths, limitations, and future directions

The strengths of our investigation include assessment of the proposed mediation model across three samples and variation in task design. Indeed, our three studies showed that bivariate relations largely replicated across male and female participants in both student and community samples. Furthermore, coping motives significantly mediated the relationship between alcohol use and an NMB across all three samples. Another methodological strength of our study is the 1-day delay, which is conducive to observing the biasing of emotional memories that occurs during the extended consolidation process (Bogie et al., 2019). Lastly, the incidental encoding of sentences provides ecological validity in reflecting everyday experiences when individuals are not actively trying to remember information. Nonetheless, the associated low overall recall performance with this approach may have limited sensitivity to detect some relations. Some procedural modifications were made to the memory task in Study 2 to increase recall performance that inherently introduced differences from Study 1. Similar patterns were observed between the female student samples in these studies; however, and identical methods were used in Studies 2 and 3.

Each study had modest sample sizes, particularly after correcting for invalid survey responses in community members; however, the pooled analysis with a larger sample supported mediation effects in females but not males. It should be noted there were about half as many student males as females in Study 2 as well as the pooled analysis, potentially limiting power for male findings. Indeed, the pattern of path coefficients in Study 2 was generally similar for male and female participants, and the individual paths were significant in the binge-drinking model for males (see Figure 2). As such, replication of the mediation models with a larger sample size and a more balanced sex distribution is warranted. Moreover, the samples were not equated in terms of demographics or the distributions of the model variables (levels of problematic alcohol use, depressive symptoms, and coping motivations), obscuring comparisons across samples. Although the patterns generally replicated, there were some differences across samples, such that depression did not play a role in the proposed model for community samples whereas it did for students. Furthermore, while we found a consistent pattern of relations between alcohol use, endorsement of coping motivations, and a self-referent NMB, the multiple analyses conducted raised the risk of Type I error. In addition, there was a low prevalence of BD episodes in all three samples of the current study, ranging from a mean of 0.5 in the male community sample to 2 episodes per month in student samples, respectively. In contrast, for example, Carbia et al. (2020) reported means of 2.2 to 2.8 BD episodes in their student samples. Finally, some procedural modifications were made to the memory task in Study 2 to increase recall performance that inherently introduced differences from Study 1. Nonetheless, similar patterns were observed between the female student samples in these studies and identical methods were used in Studies 2 and 3.

## Conclusion

Problematic drinking, whether in the form of BD or more generally, poses a problem for an individual’s emotional wellbeing and mental health; one way this materializes is through an NMB, particularly for information deemed self-referent. Results support that engaging in alcohol use as a coping mechanism for negative affect or experiencing elevated depression may explain why the NMB presents in some individuals who drink excessively but not others. Moreover, this preliminary evidence suggests that relations among alcohol use, coping motivations, and the NMB are more likely to occur in males if they have very high alcohol consumption patterns, whereas in females it occurs more consistently at moderately high levels of alcohol consumption. Future research may investigate whether the above models and observed sex/gender differences hold in clinical samples (i.e., people with co-occurring alcohol use disorder and depression diagnoses).

## Data availability statement

Study 1: The datasets presented in this article are not readily available because participants consented to data sharing under the discretion of the researchers and the institutional review board. Requests to access the datasets should be directed to the corresponding author [TAG]. Studies 2 and 3: The original contributions presented in the study are publicly available. This data can be found at: https://doi.org/10.32920/ryerson.21499290.v1.

## Ethics statement

The studies involving human participants were reviewed and approved by Toronto Metropolitan University (formerly Ryerson) Research Ethics Board. The patients/participants provided their written informed consent to participate in this study.

## Author contributions

SJ, KC, and TG designed the study and wrote and edited the article. SG collected, analyzed, and interpreted the data and designed the visualizations. All authors revised and made significant contributions to the final manuscript. All authors have approved the final article.

## Funding

Studies 2 and 3 were supported by the Natural Sciences and Engineering Research Council Undergraduate Student Research Award (564930) to SJ. The funding agency had no role in the study. Additional support was provided by a Special Projects Grant from the Faculty of Arts, Toronto Metropolitan University.

## Conflict of interest

The authors declare that the research was conducted in the absence of any commercial or financial relationships that could be construed as a potential conflict of interest.

## Publisher’s note

All claims expressed in this article are solely those of the authors and do not necessarily represent those of their affiliated organizations, or those of the publisher, the editors and the reviewers. Any product that may be evaluated in this article, or claim that may be made by its manufacturer, is not guaranteed or endorsed by the publisher.

## Footnotes

^1^ www.qualtrics.ryerson.ca

^2^ www.pavlovia.org

^3^ www.honeybeehub.com

## References

[ref1] Beck A. T. (1967). Depression: Clinical, Experimental, and Theoretical Aspects. New York: Hoeber Medical Division, Harper & Row.

[ref610] Blasberg S. A. Rogers K. H. Paulhus D. L. (2013). The Bidimensional Impression Management Index (BIMI): Measuring agentic and communal forms of impression management. J. Pers. Assess. 95, 523–531. 24328818 10.1080/00223891.2013.862252

[ref2] Blaut A. Paulewicz B. Szastok M. Prochwicz K. Koster E. (2013). Are attentional bias and memory bias for negative words causally related? J. Behav. Ther. Exp. Psychiatry 44, 293–299. doi: 10.1016/j.jbtep.2013.01.002 23411400

[ref3] Bogie J. M. Persaud M. R. Smith D. Kapczinski F. P. Frey B. N. (2019). Explicit emotional memory biases in mood disorders: a systematic review. Psychiatry Res. 278, 162–172. doi: 10.1016/j.psychres.2019.06.003 31200195

[ref4] Bone J. K. Lewis G. Roiser J. P. Blakemore S. J. Lewis G. (2021). Recall bias during adolescence: gender differences and associations with depressive symptoms. J. Affect. Disord. 282, 299–307. doi: 10.1016/j.jad.2020.12.133 33421856 PMC7615279

[ref5] Bradley B. P. Mogg K. (1994). Mood and personality in recall of positive and negative information. Behav. Res. Ther. 32, 137–141. doi: 10.1016/0005-7967(94)90095-7 8135712

[ref6] Carbia C. Corral M. Caamano-Isorna F. Cadaveira F. (2020). Emotional memory bias in binge drinking women. Drug Alcohol Depend. 209:107888. doi: 10.1016/j.drugalcdep.2020.107888 32078974

[ref7] Carpenter-Hyland E. P. Chandler L. J. (2007). Adaptive plasticity of NMDA receptors and dendritic spines: implications for enhanced vulnerability of the adolescent brain to alcohol addiction. Pharmacol. Biochem. Behav. 86, 200–208. doi: 10.1016/j.pbb.2007.01.016 17291572 PMC2662130

[ref700] Cohen B. P. Vinson D. C. (1995). Retrospective self-report of alcohol consumption: Test-retest reliability by telephone. Alcohol. Clin. Exp. Res. 19, 1156–1161. doi: 10.1111/j.1530-0277.1995.tb01595.x 8561285

[ref8] Connell A. M. Patton E. McKillop H. (2015). Binge drinking, depression, and electrocortical responses to emotional images. Psychol. Addict. Behav. 29, 673–682. doi: 10.1037/adb0000071 25915691

[ref604] Cooper M. L. Russell M. Skinner J. B. Windle M. (1992). Development and validation of a three-dimensional measure of drinking motives. Psychol. Assess. J. Consult. Clin. Psychol. 4, 123–132.

[ref605] Cooper M. L. (1994). Motivations for alcohol use among adolescents: Development and validation of a four-factor model. Psychol. Assess. 6, 117–128.

[ref9] Cox W. M. Fadardi J. S. Pothos E. M. (2006). The addiction-stroop test: theoretical considerations and procedural recommendations. Psychol. Bull. 132, 443–476. doi: 10.1037/0033-2909.132.3.443 16719569

[ref10] Decaluwe B. Fortin M. Moisan C. Muckle G. Belanger R. E. (2019). Drinking motives supporting binge drinking of Inuit adolescents. Can. J. Public Health 110, 414–421. doi: 10.17269/s41997-019-00212-5 31062338 PMC6964573

[ref11] Dir A. L. Bell R. L. Adams Z. W. Hulvershorn L. A. (2017). Gender differences in risk factors for adolescent binge drinking and implications for intervention and prevention. Front. Psych. 8:289. doi: 10.3389/fpsyt.2017.00289 PMC5743668 29312017

[ref12] Disner S. G. Beevers C. G. Haigh E. A. Beck A. T. (2011). Neural mechanisms of the cognitive model of depression. Nat. Rev. Neurosci. 12, 467–477. doi: 10.1038/nrn3027 21731066

[ref13] Disner S. G. Shumake J. D. Beevers C. G. (2017). Self-referential schemas and attentional bias predict severity and naturalistic course of depression symptoms. Cogn. Emot. 31, 632–644. doi: 10.1080/02699931.2016.1146123 26901406

[ref14] Duka T. Gentry J. Malcolm R. Ripley T. L. Borlikova G. Stephens D. N.. (2004). Consequences of multiple withdrawals from alcohol. Alcoholism 28, 233–246. doi: 10.1097/01.ALC.0000113780.41701.81 15112931

[ref15] Duyser F. A. Van Eijndhoven P. F. P. Bergman M. A. Collard R. M. Schene A. H. Tendolkar I.. (2020). Negative memory bias as a transdiagnostic cognitive marker for depression symptom severity. J. Affect. Disord. 274, 1165–1172. doi: 10.1016/j.jad.2020.05.156 32663947

[ref16] Foster D. W. Young C. M. Steers M. Quist M. C. Bryan J. L. Neighbors C. (2014). Tears in your beer: gender differences in coping drinking motives, depressive symptoms and drinking. Int. J. Ment. Health Addict. 12, 730–746. doi: 10.1007/s11469-014-9504-3 25525419 PMC4267111

[ref17] Fridrici C. Driessen M. Wingenfeld K. Kremer G. Kissler J. Beblo T. (2014). Investigating biases of attention and memory for alcohol-related and negative words in alcohol-dependents with and without major depression after day-clinic treatment. Psychiatry Res. 218, 311–318. doi: 10.1016/j.psychres.2014.03.041 24816119

[ref613] Geisner I. M. Mallett K. Kilmer J. R. (2012). An examination of depressive symptoms and drinking patterns in first year college students. Issues Ment. Health Nurs. 33, 280–287. doi: 10.3109/01612840.2011.653036 22545634 PMC3654787

[ref18] Gruhn D. (2016). An English word database of EMOtional TErms (EMOTE). Psychol. Rep. 119, 290–308. doi: 10.1177/0033294116658474 27401069

[ref19] Hammerslag L. R. Gulley J. M. (2016). Sex differences in behavior and neural development and their role in adolescent vulnerability to substance use. Behav. Brain Res. 298, 15–26. doi: 10.1016/j.bbr.2015.04.008 25882721 PMC4603997

[ref614] Hankin B. L. Abramson L. Y. (2001). Development of gender differences in depression: An elaborated cognitive vulnerability–transactional stress theory. Psychol. Bull. 127, 773–796. doi: 10.1037/0033-2909.127.6.773 11726071

[ref606] Harbke C. R. Laurent J. Catanzaro S. J. (2019). Comparison of the original and short form drinking motives questionnaire–revised with high school and underage college student drinkers. Assessment 26, 1179–1193. doi: 10.1177/1073191117731812 28938864

[ref20] Hardee J. E. Cope L. M. Munier E. C. Welsh R. C. Zucker R. A. Heitzeg M. M. (2017). Sex differences in the development of emotion circuitry in adolescents at risk for substance abuse: a longitudinal fMRI study. Soc. Cogn. Affect. Neurosci. 12, 965–975. doi: 10.1093/scan/nsx021 28338724 PMC5472107

[ref611] Hayes A. F. (2017). Introduction to Mediation, Moderation, and Conditional Process Analysis: A Regression-Based Approach. Guilford Press, New York.

[ref21] Keough M. T. O'Connor R. M. Sherry S. B. Stewart S. H. (2015). Context counts: solitary drinking explains the association between depressive symptoms and alcohol-related problems in undergraduates. Addict. Behav. 42, 216–221. doi: 10.1016/j.addbeh.2014.11.031 25486616

[ref22] Kessler K. Pajak K. M. Harkin B. Jones B. (2013). A working memory bias for alcohol-related stimuli depends on drinking score. Psychol. Addict. Behav. 27, 23–31. doi: 10.1037/a0028664 22642857

[ref23] Koob G. F. (2013). Negative reinforcement in drug addiction: the darkness within. Curr. Opin. Neurobiol. 23, 559–563. doi: 10.1016/j.conb.2013.03.011 23628232

[ref24] Kuntsche E. Knibbe R. Gmel G. Engels R. (2006). Who drinks and why? A review of socio-demographic, personality, and contextual issues behind the drinking motives in young people. Addict. Behav. 31, 1844–1857. doi: 10.1016/j.addbeh.2005.12.028 16460883

[ref25] Kuria M. W. Ndetei D. M. Obot I. S. Khasakhala L. I. Bagaka B. M. Mbugua M. N.. (2012). The association between alcohol dependence and depression before and after treatment for alcohol dependence. ISRN Psychiatry 2012:482802. doi: 10.5402/2012/482802 23738204 PMC3658562

[ref607] Lovibond P. F. Lovibond S. H. (1995). The structure of negative emotional states: comparison of the Depression Anxiety Stress Scales (DASS) with the Beck Depression and Anxiety Inventories. Behav. Res. Ther. 33, 335–343. doi: 10.1016/0005-7967(94)00075-u 7726811

[ref608] Norton P. J. (2007). Depression Anxiety and Stress Scales (DASS-21): Psychometric analysis across four racial groups. Anxiety, Stress and Coping 20, 253–265. doi: 10.1080/10615800701309279 17999228

[ref26] Olff M. (2017). Sex and gender differences in post-traumatic stress disorder: an update. Eur. J. Psychotraumatol. 8:1351204. doi: 10.1080/20008198.2017.1351204

[ref609] Osman A. Wong J. L. Bagge C. L. Freedenthal S. Gutierrez P. M. Lozano G. (2012). The Depression Anxiety Stress Scales-21 (DASS-21): Further Examination of dimensions, scale reliability, and correlates. J. Clin. Psychol. 68, 1322–1338. doi: 10.1002/jclp.21908 22930477

[ref27] Patrick M. E. Schulenberg J. E. (2011). How trajectories of reasons for alcohol use relate to trajectories of binge drinking: national panel data spanning late adolescence to early adulthood. Dev. Psychol. 47, 311–317. doi: 10.1037/a0021939 21219061 PMC3058882

[ref28] Peralta R. L. Steele J. L. Nofziger S. Rickles M. (2010). The impact of gender on binge drinking behavior among U.S. college students attending a Midwestern university: an analysis of two gender measures. Fem. Criminol. 5, 355–379. doi: 10.1177/1557085110386363

[ref29] Reed A. Carstensen L. (2012). The theory behind the age-related positivity effect [hypothesis and theory]. Front. Psychol. 3:339. doi: 10.3389/fpsyg.2012.00339 23060825 PMC3459016

[ref30] Salemink E. Wiers R. W. (2014). Alcohol-related memory associations in positive and negative affect situations: drinking motives, working memory capacity, and prospective drinking. Psychol. Addict. Behav. 28, 105–113. doi: 10.1037/a0032806 23647155

[ref31] Sanchis-Segura C. Becker J. B. (2016). Why we should consider sex (and study sex differences) in addiction research. Addict. Biol. 21, 995–1006. doi: 10.1111/adb.12382 27029841 PMC5585537

[ref601] Saunders J. B. Aasland O. G. Babor T. F. de la Puente J. R. Grant M. (1993). Development of the Alcohol Use Disorders Screening Test (AUDIT). WHO collaborative project on early detection of persons with harmful alcohol consumption. II. Addiction 88, 791–804. 8329970 10.1111/j.1360-0443.1993.tb02093.x

[ref390] Schoemann A. M. Boulton A. J. Short S. D. (2017). Determining Power and sample size for simple and complex mediation models. Soc. Psychol. Personal Sci. 8, 379–386. doi: 10.1177/1948550617715068

[ref603] Selin K. H. (2003). Test-retest reliability of the Alcohol Use Disorder Identification Test in a general population sample. Alcohol. Clin. Exp. Res. 27, 1428–1435. doi: 10.1097/01.alc.0000085633.23230.4a 14506403

[ref760] Sobell L. C. Sobell M. B. (1995). “Alcohol consumption measures,” in Assessing Alcohol Problems: A Guide for Clinicians and Researchers. eds. J.P. Allen and M. Columbus (Bethesda, MD: National Institute on Alcohol Abuse and Alcoholism), 55–73.

[ref32] Spear L. P. (2018). Effects of adolescent alcohol consumption on the brain and behaviour. Nat. Rev. Neurosci. 19, 197–214. doi: 10.1038/nrn.2018.10 29467469

[ref612] Spence J. T. Helmreich R. L. (1978). Masculinity and Femininity: Their Psychological Dimensions, Correlates, and Antecedents. Austin, TX: University of Texas Press.

[ref33] Stephens D. N. Duka T. (2008). Review. Cognitive and emotional consequences of binge drinking: role of amygdala and prefrontal cortex. Philos. Trans. R. Soc. Lond. Ser. B Biol. Sci. 363, 3169–3179. doi: 10.1098/rstb.2008.0097 18640918 PMC2607328

[ref34] Stewart S. H. Sherry S. B. Comeau M. N. Mushquash C. J. Collins P. Van Wilgenburg H. (2011). Hopelessness and excessive drinking among aboriginal adolescents: the mediating roles of depressive symptoms and drinking to Cope. Depress. Res. Treat. 2011:970169. doi: 10.1155/2011/970169 21197100 PMC3003989

[ref35] Taylor J. L. John C. H. (2004). Attentional and memory bias in persecutory delusions and depression. Psychopathology 37, 233–241. doi: 10.1159/000080719 15383713

[ref36] Tolomeo S. Macfarlane J. A. Baldacchino A. Koob G. F. Steele J. D. (2021). Alcohol binge drinking: negative and positive valence system abnormalities. Biol Psychiatry Cogn. Neurosci. Neuroimaging 6, 126–134. doi: 10.1016/j.bpsc.2020.09.010 33279457

[ref37] Tyng C. M. Amin H. U. Saad M. N. M. Malik A. S. (2017). The influences of emotion on learning and memory. Front. Psychol. 8:1454. doi: 10.3389/fpsyg.2017.01454 28883804 PMC5573739

[ref38] Ullman S. E. Relyea M. Peter-Hagene L. Vasquez A. L. (2013). Trauma histories, substance use coping, PTSD, and problem substance use among sexual assault victims. Addict. Behav. 38, 2219–2223. doi: 10.1016/j.addbeh.2013.01.027 23501138 PMC3622163

[ref39] van der Aar L. P. E. Peters S. Crone E. A. (2018). The development of self-views across adolescence: investigating self-descriptions with and without social comparison using a novel experimental paradigm. Cogn. Dev. 48, 256–270. doi: 10.1016/j.cogdev.2018.10.001

[ref40] Webb M. K. Simons J. S. Simons R. M. (2020). Affect and drinking behavior: moderating effects of involuntary attention to emotion and distress tolerance. Exp. Clin. Psychopharmacol. 28, 576–588. doi: 10.1037/pha0000329 31724419

[ref602] World Health Organization (2001). AUDIT: The Alcohol Use Disorders Identification Test: Guidelines for use in primary health care. Available at: https://www.who.int/publications/i/item/audit-the-alcohol-use-disorders-identification-test-guidelines-for-use-in-primary-health-care

[ref41] Zack M. Toneatto T. MacLeod C. M. (1999). Implicit activation of alcohol concepts by negative affective cues distinguishes between problem drinkers with high and low psychiatric distress. J. Abnorm. Psychol. 108, 518–531. doi: 10.1037//0021-843x.108.3.518 10466276

[ref42] Zack M. Toneatto T. MacLeod C. M. (2002). Anxiety and explicit alcohol-related memory in problem drinkers. Addict. Behav. 27, 331–343. doi: 10.1016/s0306-4603(01)00233-7 12118624

[ref43] Zupan Z. Žeželj I. Andjelković I. (2017). Memory bias in depression: Effects of self reference and age. J. Soc. Clin. Psychol. 36, 300–315. doi: 10.1521/jscp.2017.36.4.300

