# Supplementary material for: Binge drinking indirectly predicts a negative emotional memory bias through coping motivations and depressive symptoms: The role of sex/gender
Source: Front Psychol. 2022 Nov 21;13:998364. doi: 10.3389/fpsyg.2022.998364 (PMC9723880; doi:10.3389/fpsyg.2022.998364)
Supplement: Supplementary file 1 [file Data_Sheet_1.PDF]

****************** PROCESS for R Version 3.5 beta0.5 ******************

Written by Andrew F. Hayes, Ph.D. www.afhayes.com
Documentation available in Hayes (2018). www.guilford.com/p/hayes3

****

Model	92
Y:	Self Bias
X:	AUDIT
M1:	DMQ coping
M2:	DASS depression
W	Sex

Sample size: 292

Custom seed: 31216

****
Outcome Variable: DMQ coping
Model Summary

R	R square	MSE	F	Df1	Df2	P
0.6194	0.3836	0.611	59.75	3.00	288.0	0.0000

	coeff	se	t	p	LLCI	ULCI
constant	0.0079	0.0561	0.14	0.8886	-0.1026	0.1183
AUDIT	0.6096	0.0572	10.67	0.0000	0.4971	0.7221
Sex	-0.0079	0.0969	-0.0812	0.9353	-0.1986	0.1828

Int_1	0.0362	0.0982	0.3685	0.7128	-0.1570	0.2294

Product terms key:
Int_1 : AUDIT x sex

Test(s) of highest order unconditional interaction(s):

	R2-chng	F	df1	df2	p
X*W	0.0003	0.1358	1.0000	288.0000	0.7128

****

Outcome Variable: DASS depression

Model Summary:

R	R square	MSE	F	Df1	Df2	P
0.4112	0.1691	0.8338	11.6395	5.00	286.00	0.0000

	coeff	se	t	p	LLCI	ULCI
constant	-0.0029	0.0656	-0.0436	0.9652	-0.1319	0.1262
AUDIT	0.0658	0.0839	0.7849	0.4332	-0.0993	0.2310
DMQ Coping	0.4066	0.0833	4.8789	0.000	0.2426	0.5706
Sex	0.0029	0.1132	0.0253	0.9799	-0.2199	0.2256
Int_1	0.2793	0.1481	1.8855	0.0604	-0.0123	0.5708
Int_2	-0.4358	0.1478	-2.9485	0.0035	-0.7267	-0.1449

Product terms key:
Int_1 : AUDIT x sex
Int_2 : DMQ coping x sex

Test(s) of highest order unconditional interaction(s):

	R2-chng	F	df1	df2	p
X*W	0.0103	3.5552	1.0000	286.0000	0.0604
M1*W	0.0253	8.6938	1.0000	286.0000	0.0035

Focal predictor: AUDIT (X)
Moderator: sex (W)

Conditional effects of the focal predictor at values of the moderator(s):

sex	effect	se	t	p	LLCI	ULCI
0.0000	0.0658	0.0839	0.7849	0.4332	-0.0993	0.2310
1.0000	0.3451	0.1221	2.8273	0.0050	0.1049	0.5854

Focal predictor: DMQ coping (M1)
Moderator: sex (W)

Conditional effects of the focal predictor at values of the moderator(s):

sex	effect	se	t	p	LLCI	ULCI
0.0000	0.4066	0.0833	4.8789	0.0000	0.2426	0.5706
1.0000	-0.0292	0.1221	-0.2392	0.8111	-0.2695	0.2111

****

Outcome Variable: self bias

Model Summary:

R	R square	MSE	F	Df1	Df2	P
0.3192	0.1019	0.9076	4.6031	7.00	284.00	0.0001

	coeff	se	t	p	LLCI	ULCI
constant	-0.0005	0.0684	-0.0078	0.9938	-0.1352	0.1341
AUDIT	0.0481	0.0877	0.5482	0.5840	-0.1245	0.2206
DMQ Coping	0.0810	0.0924	0.8761	0.3817	-0.1009	0.2629
DASS Depression	0.2863	0.0771	3.7137	0.0002	0.1345	0.4380

Sex	0.0005	0.1181	0.0045	0.9964	-0.2319	0.2329
Int_1	-0.0662	0.1586	-0.4170	0.6770	-0.3784	0.2461
Int_2	0.0369	0.1574	0.2344	0.8149	-0.2729	0.3467
Int_3	-0.0886	0.1286	-0.6893	0.4912	-0.3417	0.1644

Product terms key:
Int_1 : AUDIT x sex
Int_2 : DMQ coping x sex
Int_3 : DASS depression x sex

Test(s) of highest order unconditional interaction(s):

	R2-chng	F	df1	df2	p
X*W	0.0005;	0.1739	1.0000	284.0000	0.6770
M1*W	0.0002;	0.0549	1.0000	284.0000	0.8149
M2*W	0.0015;	0.4751	1.0000	284.0000	0.4912

****

**************** DIRECT AND INDIRECT EFFECTS OF X ON Y ****************

Conditional direct effect(s) of X on Y:

sex	effect	se	t	p	LLCI	ULCI
0.0000	0.0481	0.0877	0.5482	0.5840	-0.1245	0.2206
1.0000	-0.0181	0.1322	-0.1369	0.8912	-0.2783	0.2421

Conditional indirect effects of X on Y:

INDIRECT EFFECT:
AUDIT -> DMQ coping -> self bias

sex	Effect	BootSE	BootLLCI	BootULCI
0.0000	0.0494	0.0533	-0.0503	0.1583
1.0000	0.0761	0.1066	-0.1118	0.3010

Index of moderated mediation
(differences between conditional indirect effects):

Index	BootSE	BootLLCI	BootULCI

sex 0.0267 0.1184 -0.1874 0.2784

INDIRECT EFFECT:
AUDIT -> DASS depression -> self bias

sex	Effect	BootSE	BootLLCI	BootULCI
0.0000	0.0188	0.0306	-0.0383	0.0843
1.0000	0.0682	0.0475	-0.0088	0.1760

Index of moderated mediation
(differences between conditional indirect effects):

	Index	BootSE	BootLLCI	BootULCI
sex	0.0494	0.0562	-0.0550	0.1699

INDIRECT EFFECT:
AUDIT -> DMQ coping -> DASS depression -> self bias

sex	Effect	BootSE	BootLLCI	BootULCI
0.0000	0.0710	0.0253	0.0283	0.1264
1.0000	-0.0037	0.0178	-0.0462	0.0256

Index of moderated mediation
(differences between conditional indirect effects):

	Index	BootSE	BootLLCI	BootULCI
sex	-0.0747	0.0308	-0.1447	-0.0229

******************** ANALYSIS NOTES AND ERRORS ************************

Level of confidence for all confidence intervals in output: 95

Number of bootstraps for percentile bootstrap confidence intervals: 5000

NOTE: Some cases with missing data were deleted. The number of deleted cases was: 6

****************** PROCESS for R Version 3.5 beta0.5 ******************

Written by Andrew F. Hayes, Ph.D. www.afhayes.com
Documentation available in Hayes (2018). www.guilford.com/p/hayes3

****

Model : 92
Y : self bias
X : BD
M1 : DMQ coping
M2 : DASS depression
W : sex

Sample size: 291

Custom seed: 31216

****
Outcome Variable: DMQ coping

Model Summary:

R	R square	MSE	F	Df1	Df2	P
0.3766	0.1418	0.8323	15.8127	3.00	287.0	0.0000

	coeff	se	t	p	LLCI	ULCI
constant	0.000	0.0657	-0.0007	0.9994	-0.1293	0.1292
BD	0.3812	0.0698	5.463	0.0000	0.2493	0.5185
Sex	0.0000	0.1132	0.0004	0.9997	-0.2227	0.2228
Int_1	0.0090	0.1163	0.0774	0.9383	-0.2200	0.2380

Product terms key:
Int_1 : BD x sex

Test(s) of highest order unconditional interaction(s):

	R2-chng	F	df1	df2	p
X*W	0.0000	0.0060	1.0000	287.0000	0.9383

****

Outcome Variable: DASS Depression

Model Summary:

R	R square	MSE	F	Df1	Df2	P
0.3789	0.1436	0.8507	9.5577	5.000	285.00	0.0000

	coeff	se	t	p	LLCI	ULCI
constant	-0.0082	0.0664	-0.1240	0.9014	-0.1389	0.1225
BD	-0.0636	0.0759	-0.8380	0.4027	-0.2130	0.0858
DMQ Coping	0.4576	0.0735	6.2256	0.0000	0.3129	0.6022
Sex	0.0082	0.1144	0.0720	0.9427	-0.2170	0.2334
Int_1	0.2193	0.1273	1.7225	0.0861	-0.0313	0.4699
Int_2	-0.3247	0.1259	-2.5783	0.0104	-0.5725	-0.0768

Product terms key:
Int_1 : BD x sex
Int_2 : DMQ coping x sex

Test(s) of highest order unconditional interaction(s):

	R2-chng	F	df1	df2	p
X*W	0.0089	2.9671	1.0000	285.0000	0.0861
M1*W	0.0200	6.6477	1.0000	285.0000	0.0104

Focal predictor: BD (X)
Moderator: sex (W)

Conditional effects of the focal predictor at values of the moderator(s):

sex	effect	se	t	p	LLCI	ULCI
0.0000	-0.0636	0.0759	-0.8380	0.4027	-0.2130	0.0858
1.0000	0.1557	0.1022	1.5231	0.1288	-0.0455	0.3570

Focal predictor: DMQ coping (M1)
Moderator: sex (W)

Conditional effects of the focal predictor at values of the moderator(s):

sex	effect	se	t	p	LLCI	ULCI
0.0000	0.4576	0.0735	6.2256	0.0000	0.3129	0.6022
1.0000	0.1329	0.1022	1.2998	0.1947	-0.0684	0.3341

****

Outcome Variable: self bias

Model Summary:

R	R square	MSE	F	Df1	Df2	P
0.3307	0.1094	0.9032	4.9640	7.00	283.000	0.000

	coeff	se	t	p	LLCI	ULCI
constant	0.0030	0.0684	0.0445	0.9645	-0.1316	0.1377
BD	-0.0571	0.0783	-0.7285	0.4669	-0.2113	0.0971
DMQ Coping	0.1418	0.0835	1.6974	0.0907	-0.0226	0.3062
DASS Depression	0.2899	0.0771	3.7608	0.0002	0.1382	0.4416
Sex	-0.0030	0.1322	-0.0258	0.9794	-0.2351	0.2290
Int_1	-0.0620	0.1351	-0.4687	0.6397	-0.3222	0.1983

Int_2	0.0084	0.1574	0.0621	0.9505	-0.2576	0.2743
Int_3	-0.0797	0.1262	-0.6313	0.5284	-0.3281	0.1688

Product terms key:
Int_1 : BD x sex
Int_2 : DMQ coping x sex
Int_3 : DASS Depression x sex

Test(s) of highest order unconditional interaction(s):

	R2-chng	F	df1	df2	p
X*W	0.0007	0.2197	1.0000	283.0000	0.6397
M1*W	0.0000	0.0039	1.0000	283.0000	0.9505
M2*W	0.0013	0.3985	1.0000	283.0000	0.5284

****

**************** DIRECT AND INDIRECT EFFECTS OF X ON Y ****************

Conditional direct effect(s) of X on Y:

sex	effect	se	t	p	LLCI	ULCI
0.0000	-0.0571	0.0783	-0.7285	0.4669	-0.2113	0.0971
1.0000	-0.1190	0.1065	-1.1177	0.2646	-0.3287	0.0906

Conditional indirect effects of X on Y:

INDIRECT EFFECT:

BD -> DMQ coping -> self bias

sex	Effect	BootSE	BootLLCI	BootULCI
0.0000	0.0541	0.0337	-0.0060	0.1276
1.0000	0.0586	0.0557	-0.0339	0.1878

Index of moderated mediation
(differences between conditional indirect effects):

	Index	BootSE	BootLLCI	BootULCI
sex	0.0046	0.0652	-0.1118	0.1467

INDIRECT EFFECT:

BD -> DASS depression -> self bias

sex	Effect	BootSE	BootLLCI	BootULCI
0.0000	-0.0184	0.0228	-0.0580	0.0338
1.0000	0.0327	0.0378	-0.0207	0.1263

Index of moderated mediation
(differences between conditional indirect effects):

	Index	BootSE	BootLLCI	BootULCI
sex	0.0512	0.0440	-0.0238	0.1499

INDIRECT EFFECT:

BD -> DMQ coping -> DASS depression -> self bias

sex	Effect	BootSE	BootLLCI	BootULCI
0.0000	0.0506	0.0202	0.0197	0.0980
1.0000	0.0109	0.0107	-0.0058	0.0362

Index of moderated mediation
(differences between conditional indirect effects):

	Index	BootSE	BootLLCI	BootULCI
sex	-0.0397	0.0229	-0.0915	0.0011

******************** ANALYSIS NOTES AND ERRORS ************************

Level of confidence for all confidence intervals in output: 95

Number of bootstraps for percentile bootstrap confidence intervals: 5000

NOTE: Some cases with missing data were deleted. The number of deleted cases was: 7

NOTE: Self bias was calculated as self negative sentences recalled - self positive sentences recalled, such that a higher number equals a more negative bias. Thus, positive relations indicate that as the predictor increases the negative bias increases.
